# The diagnostic ureteroscopy before radical nephroureterectomy in upper urinary tract urothelial carcinoma is not associated with higher intravesical recurrence

**DOI:** 10.1186/s12957-018-1411-9

**Published:** 2018-07-09

**Authors:** Hsiang-Ying Lee, Hsin-Chih Yeh, Wen-Jeng Wu, Jiun-Shiuan He, Chun-Nung Huang, Hung-Lung Ke, Wei-Ming Li, Chien-Feng Li, Ching-Chia Li

**Affiliations:** 1 Department of Urology, Kaohsiung Municipal Ta-Tung Hospital, Kaohsiung, Taiwan; 2 Graduate Institute of Clinical Medicine, College of Medicine, Kaohsiung Medical University, Kaohsiung, Taiwan; 3 Department of Urology, Kaohsiung Medical University Hospital, Kaohsiung, Taiwan; 4 Department of Urology, School of Medicine, College of Medicine, Kaohsiung Medical University, Kaohsiung, Taiwan; 5 Graduate Institute of Medicine, College of Medicine, Kaohsiung Medical University, No.100, Tzyou 1st Road, Kaohsiung, 807 Taiwan; 6 Department of Public Health, Kaohsiung Medical University, Kaohsiung, Taiwan; 7 Department of Urology, Ministry of Health and Welfare Pingtung Hospital, Pingtung, Taiwan; 8 Department of Pathology, Chi-Mei Medical Center, Tainan, Taiwan; 9 Department of Biotechnology, Southern Taiwan University of Science and Technology, Tainan, Taiwan; 10 National Cancer Research Institute, National Health Research Institutes, Tainan, Taiwan; 11 Institute of Clinical Medicine, Kaohsiung Medical University, Kaohsiung, Taiwan; 12 Department of Internal Medicine and Cancer Center, Kaohsiung Medical University Hospital, Kaohsiung Medical University, Kaohsiung, Taiwan

**Keywords:** Diagnostic ureteroscopy, Upper urinary tract urothelial carcinoma, Intravesical recurrence

## Abstract

**Background:**

To clarify if diagnostic ureteroscopy (URS) before radical nephroureterectomy for patients with upper tract urothelial carcinoma (UTUC) will increase the risk of intravesical recurrence.

**Methods:**

From retrospective review of cohort at our institution, 502 patients with UTUC who underwent radical nephroureterectomy with bladder cuff excision were enrolled from 1990 to 2013. Cox proportional hazards model was used to analyze the overall survival (OS), disease-free survival (DFS), metastasis-free survival (MFS), and intravesical recurrence-free survival (IVRFS). The log-rank test was used for comparing survival curves. All potential risk factors were included in the multivariate Cox proportional hazards model to recognize independent predictors. From NHI database, we included patients of UTUC without bladder cancer history using population-based database in Taiwan from 1996 to 2013. In total, 3079 URS and 2634 non-URS patients with UTUC were identified. Univariate and multivariate Cox proportional hazards regressions were performed to measure the risk of IVRFS and all-cause mortality.

**Results:**

From our database, the comparison of clinicopathological characteristics in UTUC patients between with URS biopsy group (URS+) (*n* = 206, 41%) and without URS biopsy group (URS−) (*n* = 296, 59%) was insignificantly different excluding surgical method. URS biopsy is not associated with worse OS (*p* = 0.720), DFS (*p* = 0.294), MFS (*p* = 0.808), and IVRFS (*p* = 0.560) by multivariate analysis. Only bladder cancer history is an independent significant factor to predict IVR (*p* < 0.001). The same result from NHI database, URS before radical surgery will not increase the risk of IVRFS [adjusted HR 1.136, 95% CI 1.00–1.30; *P* = 0.059] and OS [adjusted HR 0.919, 95% CI 0.82–1.04; *P* = 0.164].

**Conclusions:**

Preoperative URS manipulation is not associated with higher risk of IVRFS even in patients without bladder cancer history. Diagnostic URS is feasible to compensate the insufficient information of image in patients with UTUC.

## Background

Upper tract urothelial carcinoma (UTUC), involving renal pelvis and ureter, is rare in western countries but presents an unusual feature in Taiwan. The US National Cancer Database identifies a ratio of bladder cancer to UTUC of 93 to 7%. The male-to-female and pyelocaliceal-to-ureter tumor ratio incidence of UTUC are both about 2–3:1 [1, 2]. However, UTUC comprises up to 30% of all UCs in Taiwan, and the male-to-female ratio incidence is approximately equal, as well as in renal pelvis and ureter [3, 4]. The standard treatment of UTUC is radical nephroureterectomy with ipsilateral bladder cuff excision. However, minimally invasive uretereorenoscopic (URS) therapy in selected cases is also considered because it can preserve renal function and reduce morbidity. It also provides effective oncologic outcomes [5]. Because URS allows direct visualization of the entire collecting system, when combined with biopsies, it can increase the detection rate of UTUC lesions [6, 7]. However, pyelolymphatic, pyelotubular, and pyelovenous backflow of irrigation can occur during diagnostic URS [8, 9]. The raising about the possibility of backflow of malignant urothelial cells and tumor seeding during URS evaluation is to be considered to induce higher risk of intravesical recurrence (IVR). Although URS has been reported to be safe [10–12], more evidences are needed to establish that this procedure is not harmful for patients with UTUC. Besides, the impact of delay radical treatment because of previous URS biopsy is still controversial.

The primary recognized prognostic factors of survival for UTUC are tumor stage and grade. UTUC that invade the muscle layer usually have a relatively poor prognosis. The 5-year disease-specific survival of UTUC is < 50% for pT2/pT3 and < 10% for pT4 [13]. Based on the possibility of occult micrometastases before the surgery, metastases are often discovered after nephroureterectomy [14]. In addition, previous studies showed that after nephroureterectomy for UTUC, 25 to 69% of patients would develop a metachronous bladder tumor recurrence. Therefore, to figure out the potential risk factors of survival, metastasis and subsequent IVR for UTUC are important and will affect our clinical decision in further treatment and surveillance.

In this study, we evaluated the influence of URS biopsy on survival, metastasis, and especially focus on IVR and to analyze if delay of the curative treatment will cause worse survival. We aim to provide a more precise comparison and, therefore, also assess the impact by calculating our National Health Insurance (NHI) database. Besides, we attempted to identify the significant prognostic factors to predict disease-specific survival, metastasis-free survival, and intravesical recurrence-free survival for UTUC after nephroureterectomy.

## Methods

### Patient collection and methods of cohort in our institution

We enrolled 502 patients who underwent radical nephroureterectomy with bladder cuff excision with retrospective review of the medical records and were histologically confirmed to have UTUC from 1990 to 2013 at our institution. This study was approved by our Institutional Review Board (KMUH-IRB-20120138). None of all patients received immediate intravesical chemotherapy after radical surgery. Parameters including age, gender, smoking, bladder cancer history, estimated renal function before radical surgery, type of operation, tumor multifocality, tumor grade, pathological T stage, pathological N stage, previous diagnostic URS biopsy or none were recorded. All tumor specimens were graded by the 2004 WHO/International Society of Urologic Pathology consensus classification and staged according to the 2002 TNM classification for UCC. The decision to perform diagnostic URS or not was based on the surgeon’s judgment. The definition of metastasis progression is tumor local recurrence over tumor bed, regional lymph nodes, and distant metastasis.

Regular surveillance consisted of physical examination, cystoscopy, urine cytology, and periodic imaging studies were organized following the institutional guidelines. The schedule of cystoscopy is every 3 months for the first 2 years, every 6 months for the next 3 years, and annually thereafter. IVR was defined as pathologically identified UC in the urinary bladder after radical surgery.

### Statistical analysis of cohort in our institution

Demographic and clinicopathological factors between those with URS biopsy (URS+) and those without URS biopsy (URS−) were compared using independent sample *t* test for continuous variables and chi-square test for categorical variables. We estimated the impact of URS biopsy on overall survival (OS), disease-free survival (DFS), metastasis-free survival (MFS), and inravesical recurrence-free survival (IVRFS) by the Kaplan-Meier method. The duration of radical surgery to the cancer-specific death, metastatic progression, and intravesical recurrence or last visit was calculated to survival rates. The log-rank test was used for comparing survival curves. All potential risk factors were included in the multivariate Cox proportional hazards model to recognize independent predictors. The impact of URS biopsy was further analyzed in IVR based on tumor grade, bladder cancer history, and tumor location. In all analysis, *P* < 0.05 was considered statistically significant. Statistical analyses were performed with SPSS software, version 19 (IBM Corp., Somers, NY, USA).

### NHI database

#### Study design and data source

A longitudinal observational cohort study was conducted by using a population-based database in Taiwan from 1996 to 2013. Database contains catastrophic illness registry data, which includes most cancer, autoimmune disease, chronic psychosis, and dialysis patients. The database includes outpatients, inpatient, and enrollment data for catastrophic illness patients, so we could use the database to obtain information about patient comorbidities. All data was acquired from the National Health Insurance Research Database (NHIRD).

#### Study population

Over 99% of the 23.74 million residents of Taiwan were included in the Taiwan NHI program. Nearly one million patients are included in the Registry for catastrophic illness. UTUC patients were identified if they had a primarily UTUC diagnosis (ICD-9-CM code with 189) in inpatient hospitalization between 2000 and 2010. We exclude patients who had bladder cancer (ICD-9-CM code with 188) history before UTUC was diagnosed. All patients included received radical nephroureterectomy (ICD-9-OP 55.5). We identified patients who receive diagnostic URS (URS+) before radical nephroureterectomy as the URS cohort. Then, we identified non-URS (URS−) patients for the comparison group. All patients defined the date of receiving radical nephroureterectomy as index date. In total, 3079 URS+ and 2634 URS− patients with UTUC were identified.

#### Variable definitions

The major endpoints were to compare the risk of bladder cancer and all-cause mortality between URS and non-URS patients. Bladder cancer occurrences were identified using ICD-9 CM diagnosis code in the national catastrophic illness registry data. We defined death occurrences using the enrollment data. Several baseline characteristics were included as control variables, because they may affect outcomes. Demographic covariates included age and gender. Charlson Comorbidity Index (CCI) within 1 year before index date was used to measure patients’ baseline comorbidities. The comorbidities were diabetes mellitus (ICD-9-CM code with 250), hypertension (ICD-9-CM code with 401–405), hyperlipidemia (ICD-9-CM code with 272), ESRD (ICD-9-CM code with 585) diagnosed within 1 year before index date, and other cancers (ICD-9-CM code with 140–208) diagnosed before index date.

#### Statistical analysis

The *χ*^2^ test was used to evaluate the differences in gender, age, CCI score, and comorbidities between URS+ and URS− patients, except for mean age and mean CCI score, which were examined through independent sample *t* test. Univariate and multivariate Cox proportional hazards regressions were performed to measure the risk of bladder cancer and all-cause mortality. Hazard ratios (HRs) and 95% confidence intervals (CIs) were reported. Potential confounding variables as shown in Table 4 were controlled for multivariate models. The impact of time factors on bladder cancer incidence and UTUC cumulative survival rate was estimated with Kaplan-Meier survival curves, and differences were assessed by means of the log-rank statistic. All statistical calculations were analyzed using SAS version 9.3 (SAS institute, Cary, NC) and Stata version SE 11. A two-tailed *P* value lower than 0.05 was considered significant.

## Results

### Cohort in our institution

Table 1 lists the comparison of clinicopathological characteristics in UTUC patients between URS+ (*n* = 206, 41%) and URS− (*n* = 296, 59%). In all patients, the mean age was 65.8 years, and female (*n* = 282, 56.2%) is more than male (*n* = 220, 43.8%). One hundred five (20.9%) patients have smoking habit, and 148 (29.5%) patients have bladder cancer history. More patients (*n* = 327, 65.1%) present impaired estimated renal function, and 76 (15.1%) patients underwent dialysis before radical nephroureterectomy. The distribution of UTUC pathological T stage in this cohort was as follows: 71 (14.1%) patients had pTis-Ta, 131 (26.1%) patients had pT1, 127 (25.3%) patients had pT2, 144 (28.7%) patients had pT3, and 29 (5.8%) patients had pT4, respectively. Three hundred ninety-one (77.9%) patients have high tumor grade. Only surgical modality is the significant difference among two groups.

**Table 1:** Clinicopathological characteristics of 502 patients with upper tract urothelial carcinoma

	All patients (n=502)	URS or not		
		Yes (n=206)	No (n=296)	*p* value
	No. (%)	No. (%)	No. (%)	
Age(years)(mean [SD])	65.8 (11.0)	66.1 (10.1)	65.7 (11.6)	0.705
Gender				0.334
Male (%)	220 (43.8)	85 (41.3)	135 (45.6)	
Female (%)	282 (56.2)	121 (58.7)	161 (54.4)	
Smoking				0.984
Yes (%)	105 (20.9)	43 (20.9)	62 (20.9)	
No (%)	397 (79.1)	163 (79.1)	234 (79.1)	
Bladder cancer history				0.958
Yes (%)	148 (29.5)	61 (29.6)	87 (29.4)	
No (%)	354 (70.5)	145 (70.4)	209 (70.6)	
eGFR (mL/min/1.73 m2) (median [range])	49.4 (2.9-154.3)			0.544
≧ 60 (%)	175 (34.9)	75 (36.4)	100 (33.8)	
<60 (%)	327 (65.1)	131 (63.6)	196 (66.2)	
Dialysis				0.476
Yes (%)	76 (15.1)	34 (16.5)	42 (14.2)	
No (%)	426 (84.9)	172 (83.5)	254 (85.8)	
Surgical modality				*< 0.001
Open (%)	329 (65.5)	110 (53.4)	219 (74.0)	
Laparoscopy (%)	155 (30.9)	90 (43.7)	65 (22.0)	
Segmental resection (%)	18 (3.6)	6 (2.9)	12 (4.1)	
Tumor location				0.711
Pelvis (%)	190 (37.8)	76 (36.9)	114 (38.5)	
Ureter (%)	221 (44.0)	95 (46.1)	126 (42.6)	
Both (%)	91 (18.1)	35(17.0)	56 (18.9)	
Multifocality				0.389
Yes (%)	117 (23.3)	44 (21.4)	73 (24.7)	
No (%)	385 (76.7)	162 (78.6)	223 (75.3)	
Tumor grade				0.592
Low (%)	111 (22.1)	48 (23.2)	63 (21.3)	
High (%)	391 (77.9)	158 (76.7)	233 (78.7)	
Pathologic T stage				0.319
pTa-Tis (%)	71 (14.1)	36 (17.5)	35 (11.8)	
pT1 (%)	131 (26.1)	54 (26.2)	77 (26.0)	
pT2 (%)	127 (25.3)	47 (22.8)	80 (27.0)	
pT3 (%)	144 (28.7)	55 (26.7)	89 (30.1)	
pT4 (%)	29 (5.8)	14 (6.8)	15 (5.1)	
Pathologic N				0.999
N0 or Nx (%)	463 (92.2)	190 (92.2)	273 (92.2)	
N1-3 (%)	39 (7.8)	16 (7.8)	23 (7.8)	

*p<0.05

#### Overall survival

During mean the follow-up duration of 6.4 years, the 5-year OS rate (SD) was 89.5% (1.8) in the URS− group and 90.3% (2.2) in the URS+ group. Patients with diagnostic URS showed no negative impact on OS (*P* = 0.76) (Table 2). Excluding age, advanced T stage, higher tumor grade, lymph node involvement, and multifocality were also significantly associated with lower OS rates. Multivariate analysis showed that high tumor grade, advanced tumor T stage, and lymph node involvement were independent prognostic factors for OS [Cox regression hazard ratio (HR) 2.048, 95% CI 1.023–4.100, *P* = 0.043; HR 2.339, 95% CI 1.378–3.972, *P* = 0.002; and HR 6.342, 95% CI 3.950–10.183, *P* < 0.001, respectively; Table 2).

**Table 2:** Overall survival and disease-specific survival in univariate analysis and multivariate analysis by Cox proportional hazard model

	Overall survival				Disease-specific survival			
	Univariate analysis		Multivariate analysis		Univariate analysis		Multivariate analysis	
	HR 95% CI	*p*-value	HR 95% CI	*p*-value	HR 95% CI	*p*-value	HR 95% CI	*p*-value
Age, years	1.019 (1.002-1.036)	0.027*	1.011(0.994-1.029)	0.201	1.012 (0.993-1.032)	0.223	1.004 (0.984-1.024)	0.723
Gender (male vs female)	0.963 (0.690-1.344)	0.825	0.909 (0.633-1.393)	0.755	0.881 (0.590-1.316)	0.536	0.870(0.534-1.415)	0.574
Smoking (yes vs no)	1.073 (0.716-1.608)	0.731	0.994 (0.611-1.617)	0.981	1.182 (0.734-1.903)	0.490	0.955 (0.525-1.736)	0.880
Bladder cancer history (yes vs no)	1.041 (0.730-1.486)	0.823	0.984 (0.675-1.435)	0.932	0.928 (0.597-1.441)	0.739	0.858 (0.536-1.374)	0.523
Estimated GFR (<60 vs ≧60)	1.240 (0.865-1.777)	0.241	0.934 (0.638-1.368)	0.727	1.252 (0.809-1.937)	0.312	0.982 (0.614-1.571)	0.941
Operation method		0.098		0.184		0.078		0.101
Open	1 (reference)	-	1 (reference)	-	1 (reference)	-	1 (reference)	-
Laparoscopy	0.632 (0.415-0.963)	0.033*	0.705 (0.453-1.096)	0.121	0.558 (0.333-0.937)	0.027*	0.615 (0.356-1.063)	0.082
Segmental resection	1.028 (0.419-2.524)	0.952	1.459 (0.589-3.616)	0.414	1.141 (0.417-3.121)	0.798	1.723 (0.620-4.792)	0.297
Multifocality (yes vs no)	1.741 (1.220-2.486)	0.002*	1.319 (0.904-1.926)	0.151	1.847 (1.207-2.826)	0.005*	1.337 (0.851-2.101)	0.207
Grade (high vs low)	4.243 (2.339-7.696)	< 0.001*	2.048 (1.023-4.100)	0.043*	8.450 (3.103-23.014)	< 0.001*	2.459 (0.813-7.437)	0.111
Pathologic T stage (pT2-4 vs pTa/Tis/T1)	4.188 (2.695-6.507)	< 0.001*	2.339 (1.378-3.972)	0.002*	10.691 (4.949-23.095)	< 0.001*	5.242 (2.208-12.442)	< 0.001*
Pathologic N stage (pN1-3 vs pN0/Nx)	9.720 (6.234-15.154)	< 0.001*	6.342 (3.950-10.183)	< 0.001*	13.236 (8.144-21.510)	< 0.001*	8.084 (4.804-13.602)	< 0.001*
URS biopsy (yes vs no)	0.946 (0.664-1.348)	0.760	1.069 (0.742-1.541)	0.720	1.085 (0.717-1.644)	0.699	1.260 (0.818-1.941)	0.294

*p<0.05

#### Disease-specific survival

The DSS rate (SD) at 5 years was 92.0% (1.6) in the URS− group and 91.8% (2.0) in the URS+ group. From the univariate analysis indicated that advanced T stage, higher tumor grade, lymph node involvement, and multifocality were significantly related to lower DSS rates. In a multivariate analysis, only advanced T stage and lymph node involvement were independent risk factors of worse survival [Cox regression hazard ratio (HR) 5.242, 95% CI 2.208–12.442, *P* < 0.001; and HR 8.084, 95% CI 4.804–13.602, *P* < 0.001, respectively; (Table 2). Diagnostic URS biopsy remains not associated with worse DSS (*P* = 0.294 in multivariate analysis).

#### Metastasis-free survival

One hundred thirty-five (26.9%) patients experienced cancer progression in this cohort. The MFS rates (SD) after 5 years were 87.4% (2.0) in the URS− group and 83.8% (2.7) in the URS+ group. In multivariate analysis, multifocality, advanced T stage, and lymph node involvement were significant predictors of MFS [Cox regression hazard ratio (HR) 1.474, 95% CI 1.014–2.143, *P* = 0.042; HR 2.983, 95% CI 1.701–5.230, *P* < 0.001; and HR 5.786, 95% CI 3.696–9.058, *P* < 0.001, respectively; (Table 3). Presence of diagnostic URS biopsy was not associated with lower MFS in multivariate analysis (*P* = 0.808).

**Table 3:** Metastasis-free survival and intravesical recurrence-free survival in univariate analysis and multivariate analysis by Cox proportional hazard model

	Metastasis-free survival				Intravesical recurrence-free survival			
	Univariate analysis		Multivariate analysis		Univariate analysis		Multivariate analysis	
	HR 95% CI	*p*-value	HR 95% CI	*p*-value	HR 95% CI	*p*-value	HR 95% CI	*p*-value
Age, years	1.006 (0.990-1.022)	0.485	0.997 (0.980-1.014)	0.722	1.007 (0.991-1.023)	0.417	1.010 (0.993-1.027)	0.260
Gender (male vs female)	0.969 (0.690-1.360)	0.854	0.901 (0.598-1.357)	0.616	0.706 (0.506-0.987)	0.041*	0.721 (0.490-1.063)	0.099
Smoking (yes vs no)	1.025 (0.676-1.554)	0.908	0.850 (0.509-1.420)	0.534	1.244 (0.837-1.848)	0.281	1.068 (0.672-1.697)	0.782
Bladder cancer history (yes vs no)	0.985 (0.683-1.422)	0.936	0.969 (0.660-1.424)	0.874	4.850 (3.440-6.837)	< 0.001*	5.085 (3.571-7.241)	< 0.001*
Estimated GFR (<60 vs ≧60)	1.153 (0.802-1.658)	0.442	1.037 (0.702-1.532)	0.853	0.998 (0.703-1.417)	0.993	0.898 (0.623-1.293)	0.562
Operation method		0.711		0.854		0.527		0.991
Open	1 (reference)	-	1 (reference)	-	1 (reference)	-	1 (reference)	-
Laparoscopy	0.855 (0.582-1.258)	0.427	1.021 (0.681-1.531)	0.921	0.809 (0.549-1.192)	0.284	1.024 (0.677-1.550)	0.909
Segmental resection	0.853 (0.313-2.321)	0.755	1.336 (0.486-3.668)	0.574	1.117 (0.454-2.747)	0.809	1.041 (0.413-2.624)	0.932
Multifocality (yes vs no)	1.952 (1.366-2.789)	< 0.001*	1.474 (1.014-2.143)	0.042*	1.132 (0.765-1.676)	0.536	0.885 (0.587-1.334)	0.559
Grade (high vs low)	4.613 (2.419-8.796)	< 0.001*	1.986 (0.961-4.105)	0.064	0.981 (0.670-1.437)	0.923	1.015 (0.643-1.601)	0.950
Pathologic T stage (pT2-4 vs pTa/Tis/T1)	5.183 (3.188-8.426)	< 0.001*	2.983 (1.701-5.230)	< 0.001*	1.042 (0.743-1.462)	0.810	1.178 (0.784-1.770)	0.430
Pathologic N stage (pN1-3 vs pN0/Nx)	8.895 (5.859-13.504)	< 0.001*	5.786 (3.696-9.058)	< 0.001*	0.977 (0.428-2.231)	0.977	0.930 (0.396-2.184)	0.867
URS biopsy (yes vs no)	0.963 (0.679-1.367)	0.834	1.046 (0.727-1.505)	0.808	1.093 (0.776-1.539)	0.611	1.113 (0.776-1.596)	0.560

*p<0.05

#### Intravesical recurrence-free survival

**Fig. 1:**
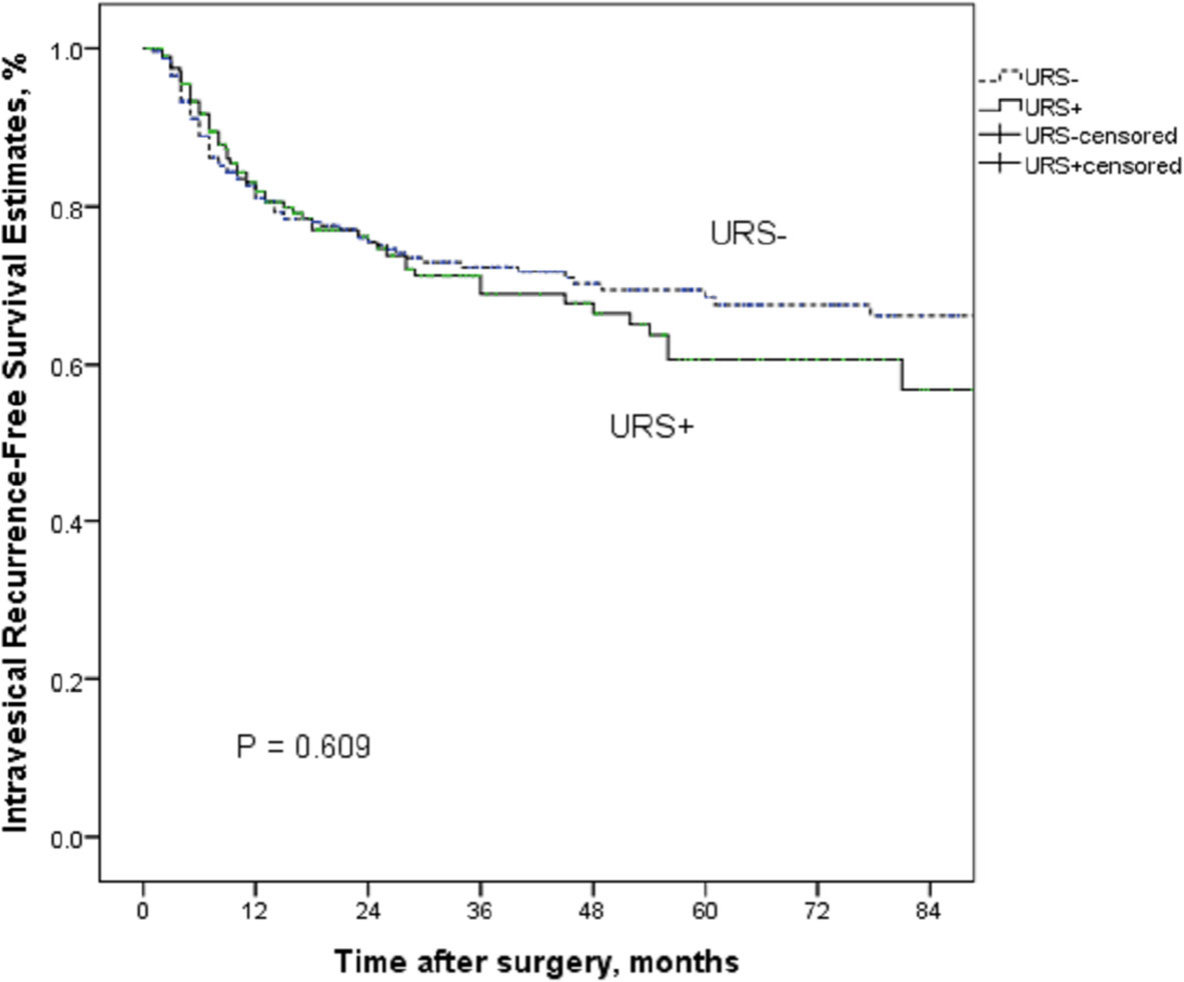
Kaplan-Meier curves for IRFS according to URS status

During the follow-up period, 138 (27.5%) patients were reported suffering from IVR. In multivariate analysis, only bladder cancer history is an independent significant factor to predict IVR (*P* < 0.001) (Table 3). Diagnostic URS biopsy performed before radical surgery did not appear to be a prognostic factor of IVR in Kaplan-Meier curves analysis (*P* = 0.609) (Fig. 1).

#### The effect of URS on IVRFS in with and without bladder cancer history

**Fig. 2:**
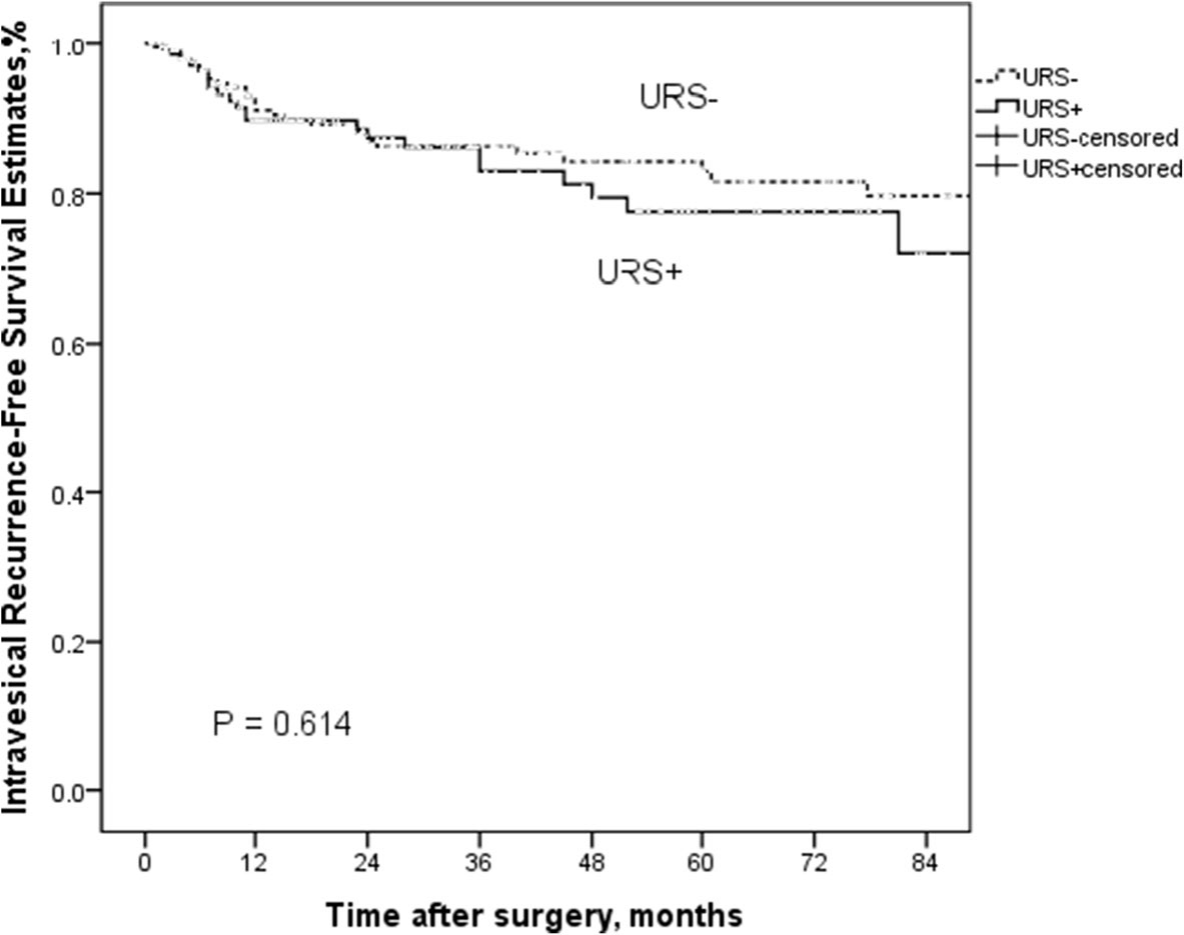
Kaplan-Meier curves for IRFS according to URS status in patients without bladder cancer history groups

**Fig. 3:**
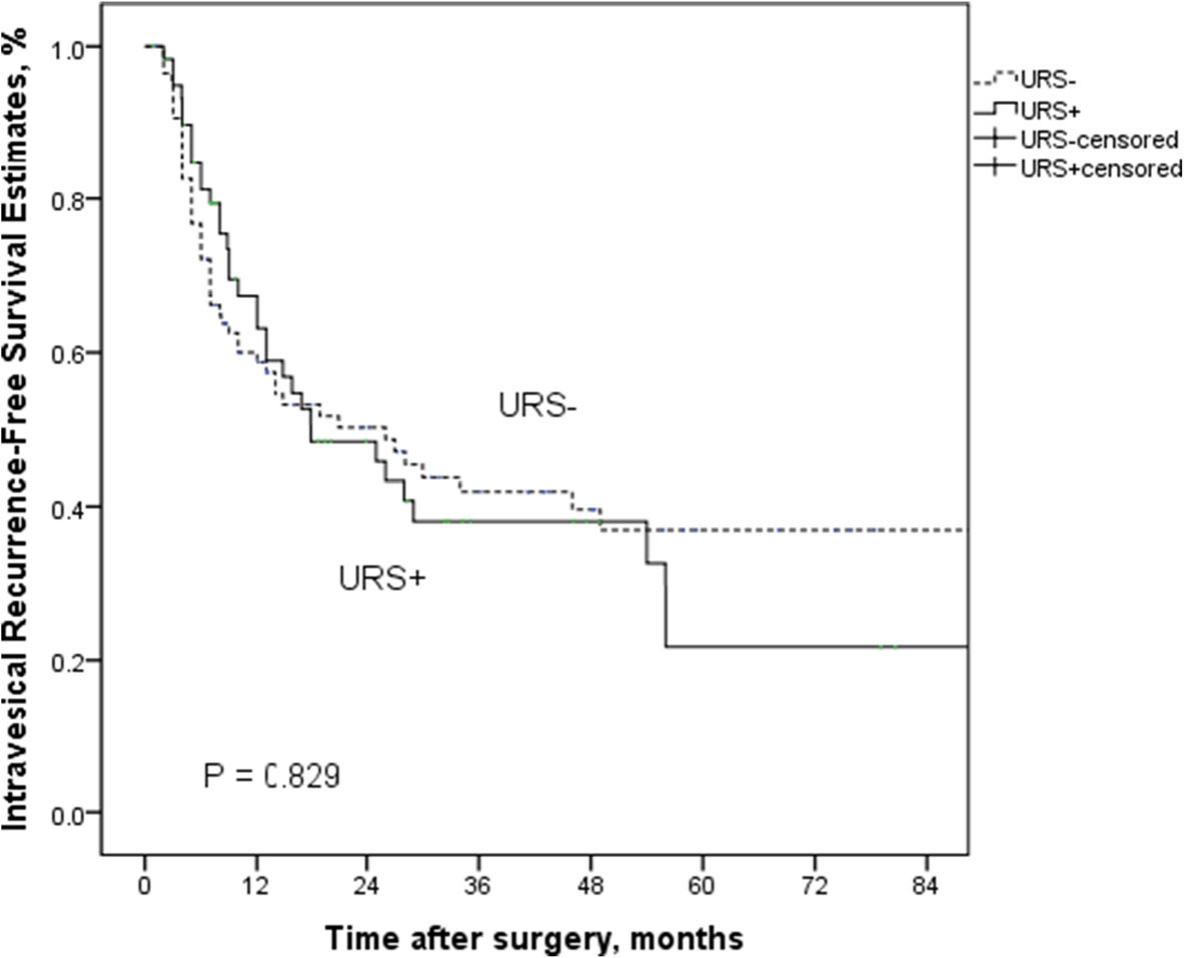
Kaplan-Meier curves for IRFS according to URS status in patients with bladder cancer history groups

In the subgroup of patients without bladder cancer history, diagnostic URS had no negative impact on IVR (*P* = 0.614) (Fig. 2). Similarly, there was no significant difference in IVRFS between the URS+ groups and URS− groups in patients with bladder cancer history (*P* = 0.829) (Fig. 3).

### NHI database

The comparison of demographics and comorbidities in UTUC patients between URS+ (*n* = 3079, 53.9%) and URS− (*n* = 2634, 46.1%) is presented in Table 4. Age and gender have similar distribution between the two groups. There were more females (*n* = 3241, 56.7%) in the study cohort. Compared with the URS− groups, significant higher prevalence of higher CCI (Charlson Comorbidity Index) score was found in URS+ (CCI ≧ 2: *n* = 2590 (84.12%) in URS+; *n* = 2048 (77.75%) in URS−, respectively).

**Table 4:** Comparison of demographics and comorbidities between with and without diagnostic ureteroscopy in patients with upper tract urothelial carcinoma

	URS+	URS-	*P*-value
	Mean±SD/ (N,%)	Mean±SD/ (N,%)	
	3,079 (53.9)	2,634 (46.1)	
Gender			
Male (N, %)	1,346 (43.72%)	1,126 (42.75%)	0.462
Female (N, %)	1,733 (56.28%)	1,508 (57.25%)	
Age (Mean±SD)	67.79 (±10.68)	67.63 (±11.15)	0.599
Age (N,%)			
<65 years	1,092 (35.47%)	952 (36.14%)	0.491
65-74 years	1,150 (37.35%)	944 (35.84%)	
>74 years	837 (27.18%)	738 (28.02%)	
CCI score (N,%)			
0	272 (8.83%)	341 (12.95%)	<0.001*
1	217 (7.05%)	245 (9.30%)	
≧2	2,590 (84.12%)	2,048 (77.75%)	
Comorbidity			
Hypertension			
No	1,443 (46.87%)	1,300 (49.35%)	0.061
Yes	1,636 (53.13%)	1,334 (50.65%)	
Hyperlipidemia			
No	2,371 (77.01%)	2,138 (81.17%)	<0.001*
Yes	708 (22.99%)	496 (18.83%)	
Diabetes			
No	2,345 (76.16%)	2,054 (77.98%)	0.103
Yes	734 (23.84%)	580 (22.02%)	
ESRD			
No	2,490 (80.87%)	2,072 (78.66%)	0.038*
Yes	589 (19.13%)	562 (21.34%)	

*CCI* Charlson Comorbidity Index, *ESRD* End-stage renal disease

*p<0.05

#### Intravesical recurrence-free survival

**Fig. 4:**
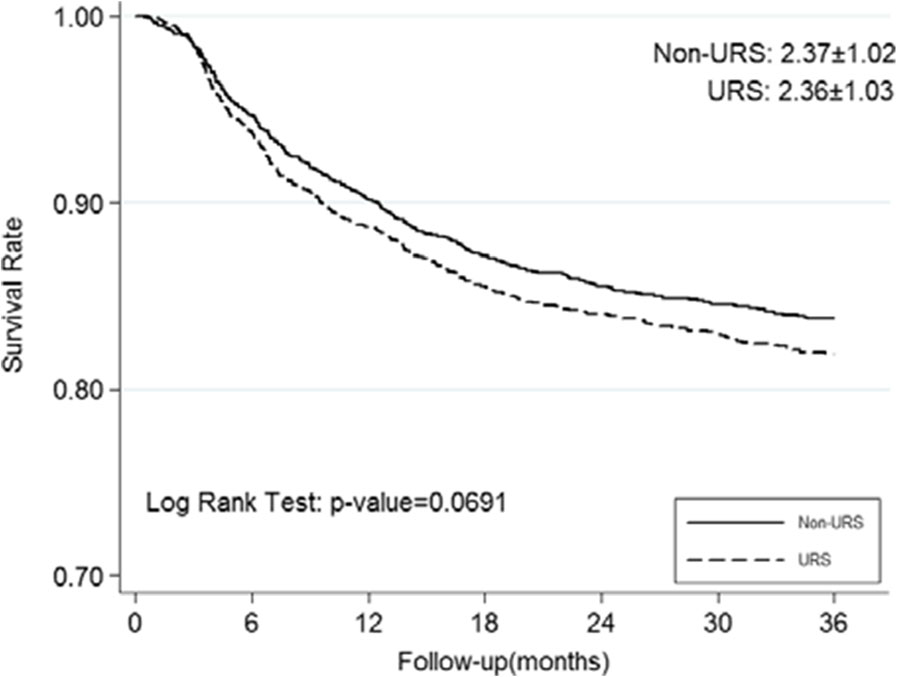
Kaplan-Meier curves for IRFS according to URS status in NHI database

The overall incidences of IVR were 62.79 and 70.92 per 1000 person-years in the URS− and URS+ cohorts, respectively, shown in Table 5, Fig. 4. According to multivariable Cox proportional hazard regression analysis, URS+ did not have a significantly higher risk of IVR [adjusted HR 1.136, 95% CI 1.00–1.30; *P* = 0.059]. Male patients and the patients with ESRD revealed significant higher risk of IVR [adjusted HR 1.293, 95% CI 1.13–1.48; *P* < 0.001 and HR 1.221, 95% CI 1.04–1.44; *P* = 0.017, respectively].

**Table 5:** Cox models measured incidence densities and hazard ratio of intravesical recurrence outcome

	N	Total person-year	Case	per 1000 person-year Incident rate	Crude HR (95% CI)	*p*-value	adjust HR (95% CI)	*p*-value
Main Effect								
URS- (Ref.)	2,634	6,243	392 (14.88%)	62.79	1 (Ref.)		1 (Ref.)	
URS+	3,079	7,261	515 (16.73%)	70.92	1.129 (0.99 - 1.29)	0.069	1.136 (1.00 - 1.30)	0.059
Baseline Patient Demographic Characteristics								
Gender								
Female (Ref.)	3,241	7,798	464 (14.32%)	59.50	1 (Ref.)		1 (Ref.)	
Male	2,472	5,707	443 (17.92%)	77.63	1.285 (1.13 - 1.46)	<0.001*	1.293 (1.13 - 1.48)	<0.001*
Age Categories								
<65 yr(Ref.)	2,044	5,028	332 (16.24%)	66.03	1 (Ref.)		1 (Ref.)	
65-74 yr	2,094	4,994	324 (15.47%)	64.87	0.978 (0.84 - 1.14)	0.771	0.994 (0.85 - 1.16)	0.935
>74yr	1,575	3,482	251 (15.94%)	72.09	1.067 (0.91 - 1.26)	0.440	1.087 (0.92 - 1.29)	0.334
CCI score Categories								
0 (Ref.)	613	1,467	95 (15.50%)	64.75	1 (Ref.)		1 (Ref.)	
1	462	1,105	68 (14.72%)	61.53	0.947 (0.69 - 1.29)	0.731	0.932 (0.68 - 1.28)	0.661
2+	4,638	10,932	744 (16.04%)	68.06	1.048 (0.85 - 1.30)	0.667	0.917 (0.73 - 1.15)	0.454
Hypertension								
No (Ref.)	2,743	6,560	437 (15.93%)	66.61	1 (Ref.)		1 (Ref.)	
Yes	2,970	6,944	470 (15.82%)	67.68	1.009 (0.89 - 1.15)	0.887	1.011 (0.88 - 1.16)	0.870
Hyperlipidemia								
No (Ref.)	4,509	10,591	731 (16.21%)	69.02	1 (Ref.)		1 (Ref.)	
Yes	1,204	2,913	176 (14.62%)	60.42	0.884 (0.75 - 1.04)	0.141	0.883 (0.74 - 1.05)	0.155
DM								
No (Ref.)	4,399	10,447	686 (15.59%)	65.66	1 (Ref.)		1 (Ref.)	
Yes	1,314	3,057	221 (16.82%)	72.29	1.098 (0.94 - 1.28)	0.227	1.152 (0.98 - 1.35)	0.085
ESRD								
No (Ref.)	4,562	10,876	704 (15.43%)	64.73	1 (Ref.)		1 (Ref.)	
Yes	1,151	2,628	203 (17.64%)	77.23	1.186 (1.01 - 1.39)	0.033*	1.221 (1.04 - 1.44)	0.017*

*Crude HR* relative hazard ratio, *Adjusted HR* adjusted hazard ratio controlling for age, gender, CCI score, Hypertension, Hyperlipidemia, diabetes and ESRD

*p<0.05

#### Overall survival

**Fig. 5:**
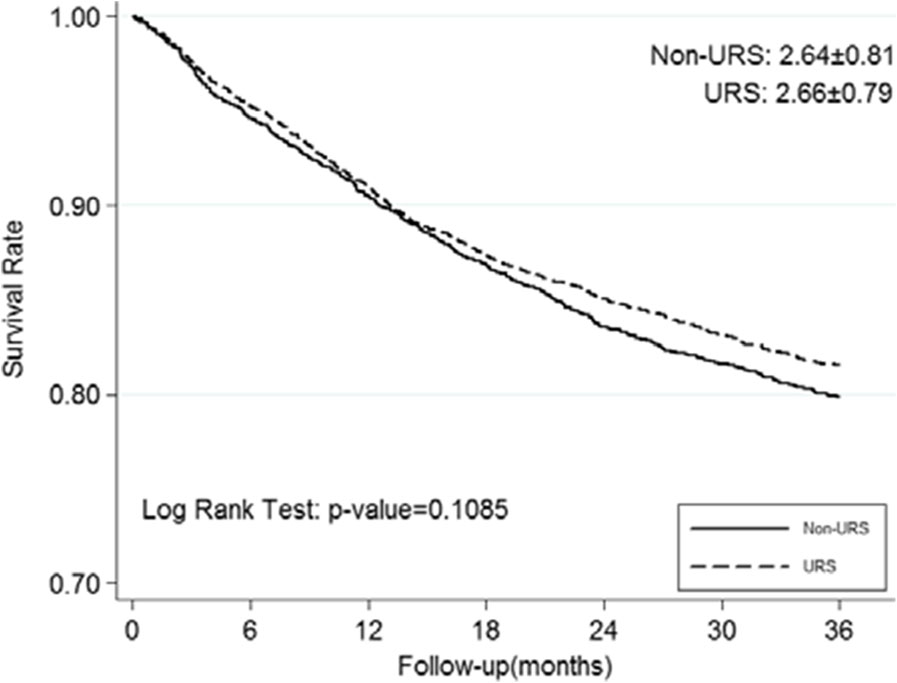
Kaplan-Meier curves for overall survival according to URS status in NHI database

The incidence mortality rates were 76.50 and 69.31 per 1000 person-years in the URS− and URS+ cohorts, respectively (Table 6, Fig. 5). Compared with URS−, URS+ groups also have no negative impact on OS [adjusted HR 0.919, 95% CI 0.82–1.04; *P* = 0.164]. Higher mortality is found in male patients, more aged patients, and ESRD patients [adjusted HR 1.225, 95% CI 1.09–1.38, *P* = 0.001; > 74 years: HR 2.290, 95% CI 1.96–2.68, *P* < 0.001; HR 1.254, 95% CI 1.08–1.46, *P* = 0.003, respectively].

**Table 6:** Cox models measured incidence densities and hazard ratio of overall survival

	N	Total person-year	Case	per 1000 person-year Incident rate	Crude HR (95% CI)	*p*-value	adjust HR (95% CI)	*p*-value
Main Effect								
URS- (Ref.)	2,634	6,941	531 (20.16%)	76.50	1 (Ref.)		1 (Ref.)	
URS+	3,079	8,195	568 (18.45%)	69.31	0.907 (0.81 – 1.02)	0.108	0.919 (0.82 – 1.04)	0.164
Baseline Patient Demographic Characteristics								
Gender								
Female (Ref.)	3,241	8,643	579 (17.86%)	66.99	1 (Ref.)		1 (Ref.)	
Male	2,472	6,493	520 (21.04%)	80.08	1.191 (1.06 – 1.34)	0.004*	1.225 (1.09 - 1.38)	0.001*
Age Categories								
<65 yr(Ref.)	2,044	5,647	266 (13.01%)	47.10	1 (Ref.)		1 (Ref.)	
65-74 yr	2,094	5,546	410 (19.58%)	73.93	1.561 (1.34 - 1.82)	<0.001*	1.597 (1.36 - 1.87)	<0.001*
>74yr	1,575	3,943	423 (26.86%)	107.28	2.245 (1.93 - 2.62)	<0.001*	2.290 (1.96 – 2.68)	<0.001*
CCI score Categories								
0 (Ref.)	613	1,637	109 (17.78%)	66.57	1 (Ref.)		1 (Ref.)	
1	462	1,209	98 (21.21%)	81.03	1.211 (0.92 – 1.59)	0.169	1.134 (0.86 – 1.49)	0.372
2+	4,638	12,289	892 (19.23%)	72.58	1.088 (0.89 – 1.33)	0.407	0.956 (0.78 – 1.18)	0.671
Hypertension								
No (Ref.)	2,743	7,322	504 (18.37%)	68.83	1 (Ref.)		1 (Ref.)	
Yes	2,970	7,814	595 (20.03%)	76.15	1.103 (0.98 – 1.24)	0.104	0.992 (0.88 – 1.12)	0.896
Hyperlipidemia								
No (Ref.)	4,509	11,883	908 (20.14%)	76.41	1 (Ref.)		1 (Ref.)	
Yes	1,204	3,253	191 (15.86%)	58.72	0.772 (0.66 – 0.90)	0.001*	0.777 (0.66 – 0.91)	0.002*
DM								
No (Ref.)	4,399	11,682	825 (18.75%)	70.62	1 (Ref.)		1 (Ref.)	
Yes	1,314	3,454	274 (20.85%)	79.32	1.122 (0.98 - 1.29)	0.099	1.141 (0.99 - 1.32)	0.072
ESRD								
No (Ref.)	4,562	12,134	862 (18.90%)	71.04	1 (Ref.)		1 (Ref.)	
Yes	1,151	3,002	237 (20.59%)	78.95	1.110 (0.96 - 1.28)	0.153	1.254 (1.08 - 1.46)	0.003*

*Crude HR* relative hazard ratio, *Adjusted HR* adjusted hazard ratio controlling for age, gender, CCI score, Hypertension, Hyperlipidemia, diabetes and ESRD

*p<0.05

#### Intravesical recurrence-free survival among subgroups from our cohort

**Fig. 6:**
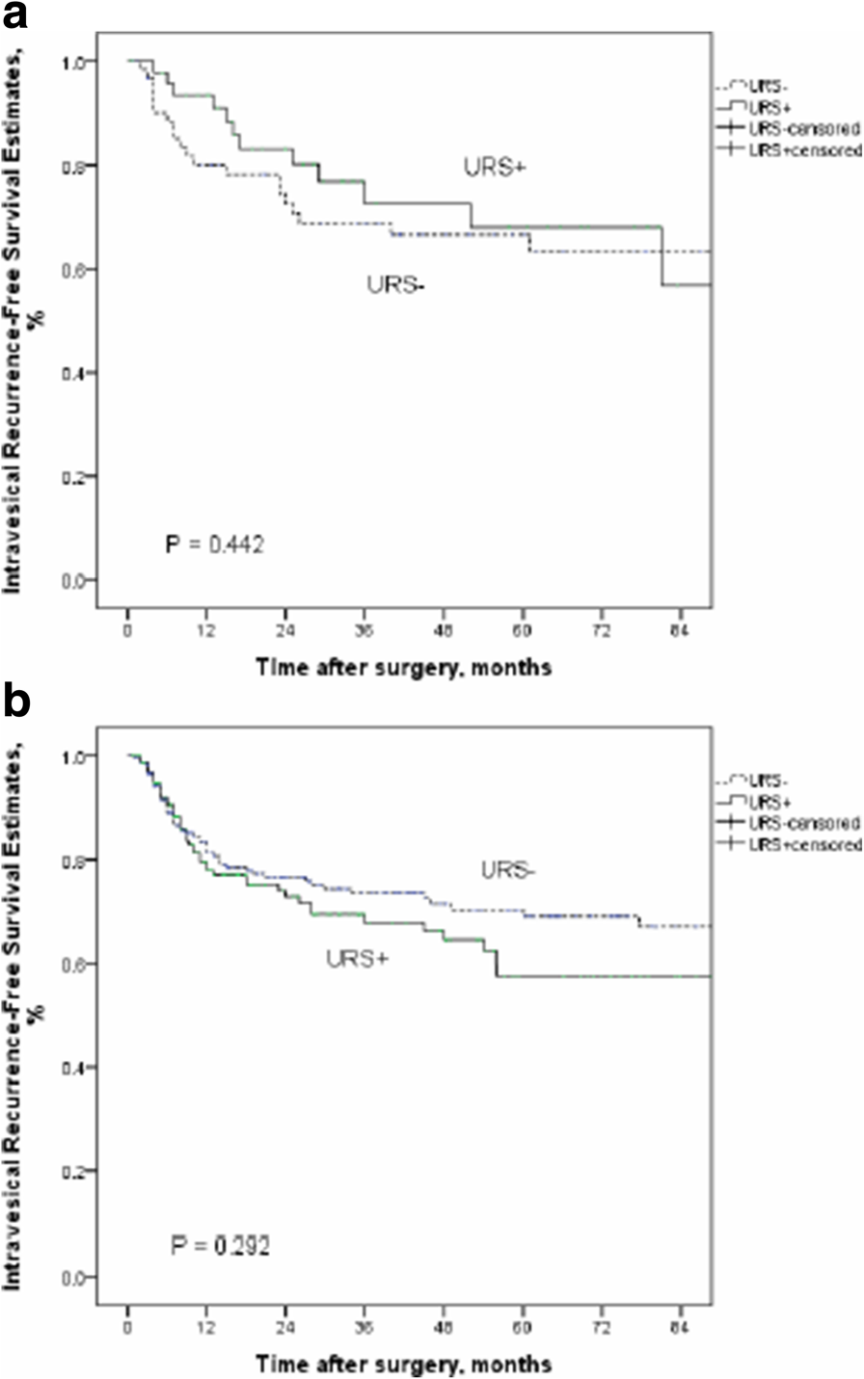
Kaplan-Meier curves for IRFS according to URS status in low grade groups (**a**) and high grade groups (**b**)

**Fig. 7:**
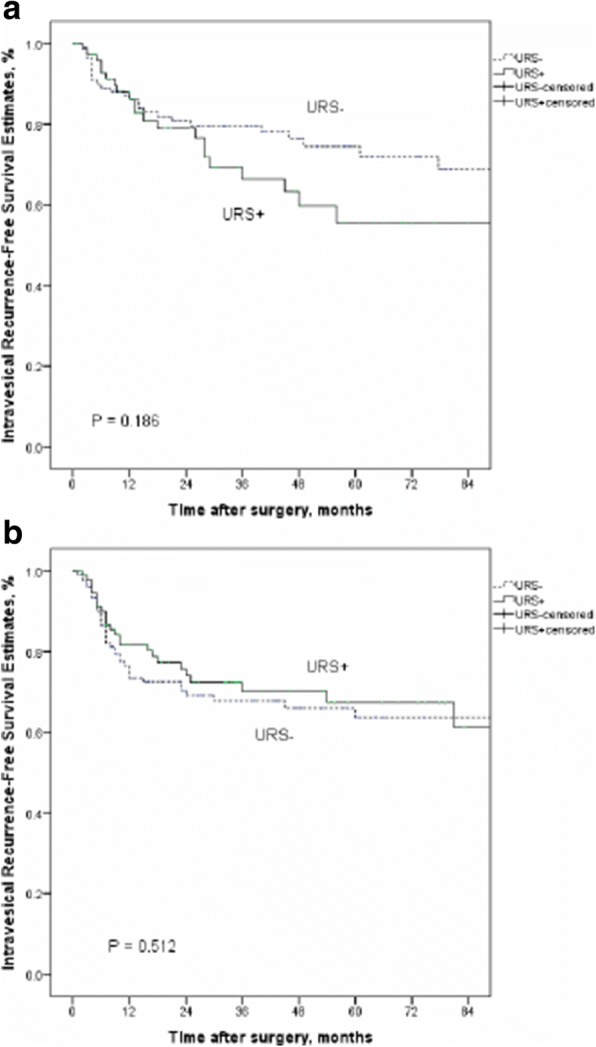
Kaplan-Meier curves for IRFS according to URS status in renal pelvis location groups (**a**) and ureter location groups (**b**)

Among our subgroups of high grade and low grade, there is no significant difference between patients with URS and without URS biopsy (*P* = 0.442 in low grade; *P* = 0.292 in high grade, respectively) (Fig. 6a, b). Compared with our data, no matter where the tumor location is, URS biopsy before radical surgery do not enhance the risk of IVR (*P* = 0.186 in renal pelvis location; *P* = 0.512 in ureter location, respectively) (Fig. 7a, b).

## Discussion

Because pathological T stage and tumor grade have been established as major prognostic factors for UTUC, it is important to determine tumor architecture, grade, and stage assessment before definite treatment [15]. To compensate for the limitations of a cross-sectional image study, URS can be used as a direct visualization method for diagnosis especially combined with biopsies. Based on the analysis of previous studies, URS has been shown to have significantly higher accuracy, specificity, and positive predictive value than multiphase computed tomography urography (MCTU) [16]; however as many hospitals do not have MCTU equipment, URS evaluation is even more important. Advance in endourologic technologies improve diagnostic accuracy without severe adverse effects and have been increasingly used for treatment purposes by direct tumor ablation with laser in selected patients. However, some previous studies have raised concerns about the possibility of intraluminal tumor seeding with manipulation during ureteroscopy and an increased incidence of IVR and metastasis [17, 18]. On the other hand, delaying radical nephroureterectomy may reduce survival rate. In order to clarify these issues and the conflicting results from several previous studies, we used both our own database and the National Health Insurance Research database to analyze the impact of pre-radical nephroureterectomy ureteroscopy on survival rate, metastasis rate, and especially the IVR rate.

Our cohort study revealed that bladder cancer history was the only risk factor for IVR after nephroureterectomy on multivariate Cox regression analysis, which is similar to the results of previous studies [19, 20]. We found that diagnostic URS did not increase intravesical recurrence rate. In accordance with the treatment guidelines at our institution, we arranged for regular imaging studies for clinical staging before diagnostic procedure or radical surgery. For patients who had a large and obvious tumor, we considered performing radical surgery without URS biopsy. For pathology evaluation before radical surgery, we performed not only URS inspection but also simultaneous tumor biopsy. We further analyzed the subgroups of patients with and without a history of bladder cancer and noted no increase in the risk of IVR whether or not patients underwent URS before radical surgery. From the NHI database results, we included only patients with UTUC who had no previous or concurrent bladder cancer history, which is a well-known important predictive factor for IVR. This analysis also revealed that diagnostic URS was not significantly associated with increased IVR. In addition, we compared the duration of nephroureterectomy to bladder cancer recurrence between the groups with and without URSs and found that the URS group did not have significantly accelerated IVR.

Although the use of diagnostic URS followed by nephroureterectomy may raise concerns about delaying the course of curative treatment in patients with UTUC, no impact was noted on cancer-specific, bladder, or contralateral upper urinary tract recurrence and metastasis-free survival [21]. Similar to our findings, we still maintain a regular follow-up schedule based on standard guidelines instead of reducing the follow-up interval even when using diagnostic URS before radical surgery.

Early diagnosis of UTUC is still a challenging issue, especially for lower stage or flat growth pattern tumors. Although various imaging modalities are available including computed tomography urography which replaced intravenous urography owing to its higher detection rate, CT urography often cannot be used to identify carcinoma in situ or to localize superficial extensions of the tumor. Chronic inflammation can easily mimic urothelial cancer, leading to false-positive findings, which constitute a limitation of CT urography for the diagnosis of UTUC [22]. Liquid biopsy is also a popular continuing research target [23]. In addition, endoscopic management or kidney-sparing surgery can be considered for a specialized group of low-risk patients with impaired renal function; therefore, in order to avoid an unnecessary radical surgery, the imperative role of URS biopsy cannot be neglected. At a minimum, it should be included as one multimodality diagnostic option. Cutress et al. reported an analysis comparing endoscopic and laparoscopic management of noninvasive UTUC, and endoscopic treatment may provide non-inferior disease-free survival compared to radical surgery only in lower grade disease [24]. In their study, they found a significantly higher risk of IVR when patients received endoscopic management rather than laparoscopic management in higher grade (G2 and G3) disease but not in low-grade disease. Among our high-grade and low-grade subgroups, there was no significant difference between patients with and without URS biopsy. Moreover, in a previous study, about 3% of patients with suspected UTUC who underwent radical surgery were reported to eventually have benign pathology and that they need to be prevented from unnecessary radical surgery [25]. Therefore, careful investigation in patients with previously suspected UTUC is critical.

In previous studies, about half of patients with UTUC encountered IVR after receiving radical surgery [26], which is higher than our results (around 27% (138/502)). Although IVR was not related to an increased risk of poor survival or distant metastasis, about 5–10% of recurrent bladder tumors progressed to a muscle-invasive state that is an important risk factor for poor survival and metastasis [27]. Given the high risk of IVR, patients with UTUC after undergoing radical surgery are recommended to undergo regular endoscopic surveillance. Therefore, it is important to consider this issue to reduce the risk of IVR. Our current study indicates that bladder tumor history is the only prognostic factor for IVR. If a higher probability of IVR is suspected after radical surgery, single-dose immediate intravesical chemotherapy is reported to be a feasible and safe strategy to prevent IVR in patients with UTUC [28]. Yoo et al. hypothesized that tumor location is a key factor affecting IVR after URS with manipulation. In their assessment, the reason for bladder tumor recurrence from the ureter tumor is the previous shedding of tumor cells owing to the short distance from the ureter tumor to the bladder. They concluded that URS biopsy was an independent risk factor for IVR only in patients with a renal pelvis tumor which offsets the protective distance between the renal pelvis and the bladder [29]. Compared to our data, no matter where the tumor is located, URS biopsy before radical surgery does not increase the risk of IVR. Based on these results, the actual mechanism of IVR is still not clear; it may not be simply from tumor detachment induced by manipulation.

There are some limitations to the present study in addition to its retrospective design. However, in order to increase its accuracy, we also analyze the National Taiwan Insurance Database and included more patients with UTUC. The first limitation was that some possible prognostic factors for IVR including bladder cuff management method and concomitant carcinoma in situ were not included. Furthermore, we could not compare our patients with those receiving URS without biopsy or with laser treatment for kidney-sparing surgery. In addition, the impact of URS on the conditional survival of IVR over time needs to be assessed because Shigeta et al. concluded that the influence of most predictive factors for IVR diminish over time [30]. A recent meta-analysis comprising six studies concluded that diagnostic URS before radical surgery seems to increase the risk of IVR after radical surgery. However, all of these six studies had the same results, and they need to be considered cautiously with various limitations. As we discussed, previous analyses have reported that diagnostic URS before radical surgery has no significant effect on IVR. On the other hand, the duration required to define bladder tumor recurrence and a primary bladder tumor event is still unclear. Nevertheless, our findings indicate that a two-session approach was not an independent risk factor for increased IVR.

## Conclusion

Diagnostic URS before radical nephroureterectomy does not significantly increase the risk of worse survival, progression, and intravesical recurrence even in patients who have no history of bladder cancer. Our data from both our institution and the NHI database indicate that diagnostic URS can be part of a diagnostic strategy especially in flat, small tumors which are difficult to identify on imaging studies and when patients plan to undergo conservative treatment.
